# Optical cryptography with biometrics for multi-depth objects

**DOI:** 10.1038/s41598-017-12946-8

**Published:** 2017-10-11

**Authors:** Aimin Yan, Yang Wei, Zhijuan Hu, Jingtao Zhang, Peter Wai Ming Tsang, Ting-Chung Poon

**Affiliations:** 1 Key Laboratory of Optoelectronic Material and Device, College of Mathematics and Science, Shanghai Normal University, Shanghai, 200234 China; 2 Department of Electronic Engineering, City University of Hong Kong, Hong Kong, SAR China; 3 Bradley Department of Electrical and Computer Engineering, Virginia Tech, Blacksburg, VA 24061 USA

## Abstract

We propose an optical cryptosystem for encrypting images of multi-depth objects based on the combination of optical heterodyne technique and fingerprint keys. Optical heterodyning requires two optical beams to be mixed. For encryption, each optical beam is modulated by an optical mask containing either the fingerprint of the person who is sending, or receiving the image. The pair of optical masks are taken as the encryption keys. Subsequently, the two beams are used to scan over a multi-depth 3-D object to obtain an encrypted hologram. During the decryption process, each sectional image of the 3-D object is recovered by convolving its encrypted hologram (through numerical computation) with the encrypted hologram of a pinhole image that is positioned at the same depth as the sectional image. Our proposed method has three major advantages. First, the lost-key situation can be avoided with the use of fingerprints as the encryption keys. Second, the method can be applied to encrypt 3-D images for subsequent decrypted sectional images. Third, since optical heterodyning scanning is employed to encrypt a 3-D object, the optical system is incoherent, resulting in negligible amount of speckle noise upon decryption. To the best of our knowledge, this is the first time optical cryptography of 3-D object images has been demonstrated in an incoherent optical system with biometric keys.

## Introduction

Information security has become a practical and serious issue with the increasing growth of internet and telecommunications. Optical information encryption techniques have attracted the interest of many researchers because of their unique advantages, such as multi-dimensional capability^1,2^. Since double random phase encoding (DRPE)^3^ was proposed, many encryption methods, such as fractional Fourier transform^4^, Fresnel transform^5^, digital holography^6^ and polarization^7^, have been further developed in order to enhance cryptosystem security. However, DRPE has been found to be quite vulnerable^8,9^. Recently, optical asymmetric cryptosystems, such as phase-truncated fractional Fourier transform^10^ and Yang-Gu algorithm^11^, have been proposed to solve the inherent issue in symmetric cryptosystems. Optical asymmetric key cryptosystems break the linearity of the DRPE technique and make the security system more reliable. Subsequently, asymmetric cryptosystems based on gyrator wavelet transform, fractional Fourier transform and joint transform correlator architecture^12–14^ have been developed and optical cryptosystem security is further improved.

Biometric information authentication is also emerging as an important research field in the domain of optical security. Tashima *et al*.^15^ and Takeda *et al*.^16^ have proposed the encryption methods using fingerprint keys with DRPE to avoid some attacks and improve security. In traditional cryptography, key is not strongly linked with its owner. This results in difficulty for the user to remember a long decryption key or in the situation where the private key is lost and hence a new set of private and public keys have to be generated again in the case of asymmetrical cryptography. Biometrics, such as fingerprint, face and iris, is one of the most trustworthy concerns with high degree of assurance for person verification. Hence, researchers are trying to integrate biometrics with cryptography. However, most of the biometric authentication techniques are geared towards encrypting 2-D information such as image and digital data. Practically, there is a growing demand to utilize 3-D information of the object with the advent of 3-D imaging. For example, 3-D information can be encrypted by use of digital holography^17–20^. But it is difficult to encrypt a large 3-D object by conventional digital holography because of the finite size of pixels in a recording CCD camera^21^. Chen *et al*.^22^ have demonstrated asymmetric cryptography using 3-D space-based model, and it was shown that conventional 2-D processing can be converted into 3-D space. Yang *et al*.^23^ introduced an encryption algorithm for 3-D information using optical asymmetric keys and digital interferometry. However, most of the optical encryption techniques have been coherent optical techniques which inherently have poor signal-to-noise ratio (S/N) compared to their incoherent counterparts^24^. Poon *et al*.^25^ have proposed optical scanning cryptography (OSC) to encrypt information incoherently based on optical scanning holography (OSH)^26^. Its first experiment has been demonstrated recently together with biometric encryption and decryption^9^. Although the S/N of the decrypted image is enhanced, the method has been developed only to the encryption of 2-D planar images.

In this work, we propose novel optical cryptography with biometric keys for encrypting multi-depth 3-D objects. The proposed system is also incoherent, meaning speckles noise is absent from its encryption and decryption of the 3-D object. To our knowledge, it is the first time an optical encryption system with such capabilities is successfully developed and reported. Organization of the paper is given as follows. In the next section, the framework and theory of our cryptographic system are described. Subsequently, we shall describe our proposed method for encrypting 3-D object images based on the cryptographic framework. Next, experimental results are shown to demonstrate the feasibility of our approach, and finally in the last section, we make some concluding remarks.

## General theory on proposed cryptosystem

**Figure 1:**
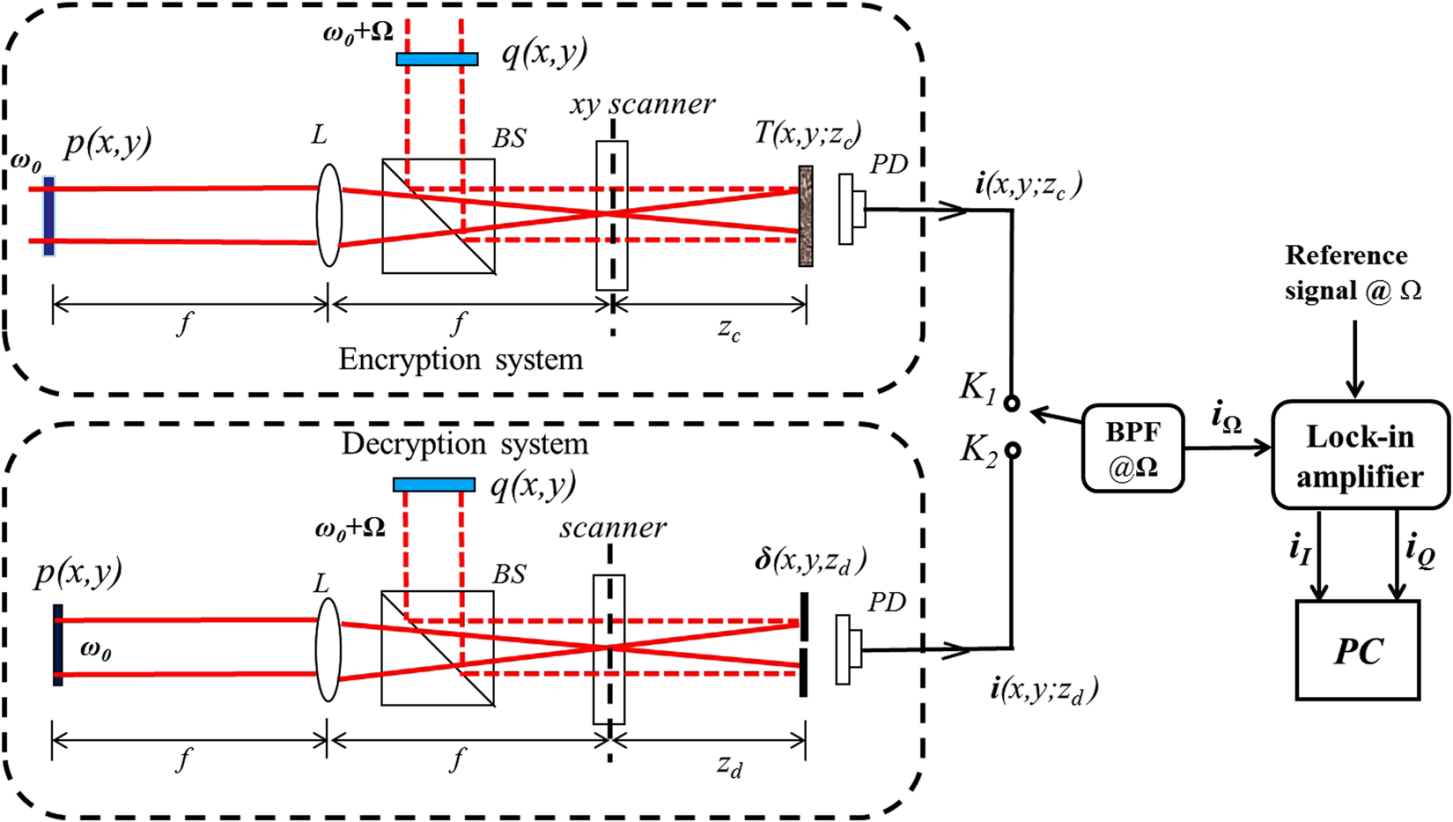
Overall cryptosystem: BPF@Ω is an electronic bandpass filter tuned at frequency Ω.

In Fig. 1, we show an overall cryptosystem, where the optical system on the top presents a subsystem of optical encoding or encryption when the switch is at *K*_1_, and the optical system on the bottom shows an optical subsystem for decryption when the switch is at *K*_2_. Note that the two subsystems basically are the same except that in the encryption system, 3-D object image of complex amplitude *T*(*x*, *y*; *z*_*c*_) to be encrypted, at coding distance *z*_*c*_ away from the *xy* scanner, is scanned with the switch at *K*_1_, whereas in the decryption system, a pin hole, *δ*(*x*, *y*; *z*_*d*_), at *z*_*d*_ away from the *xy* scanner, is scanned when the switch is at *K*_2_. The parameter *z*_*d*_ is referred to as the ‘decoding distance’. In what follows, we discuss the general principle of the optical system.

### Encryption theory

In the encryption system shown in Fig. 1, we have two encoding masks, *p*(*x*, *y*) and *q*(*x*, *y*). In practice, they can be loaded on spatial light modulators in the optical system. *p*(*x*, *y*) and *q*(*x*, *y*) are illuminated by plane waves at temporal frequencies ω_0_ and ω_0_ + Ω, respectively. The two fields after the two masks are combined by beamsplitter (*BS*) and projected onto input represented by complex amplitude distribution *T*(*x*, *y*; *z*_*c*_), which is located 2 *f* + *z*_*c*_ away from *p*(*x*, *y*) with *f* being the focal length of Lens *L*. Again, *z*_*c*_ is the coding distance. The distance from *q*(*x*, *y*) to the input is given by *z*_*q*_ = *z*_*q0*_ + *z*_*c*_. The input is 2-D scanned by the combination of the two fields and this can be done, for example, by projecting the combined optical beams through an *xy* mirror-scanner onto the input, which is shown in Fig. 1. In the system, we have utilized different transforms for the two different encoding masks in order to add complexity to the overall system. We have Fresnel transform of *q*(*x*, *y*) along one optical path and then on the other optical path, we have Fourier transform of *p*(*x*, *y*), that is the spectrum of *p*(*x*, *y*) through Lens *L*. Therefore, the use of Fresnel transform of *q*(x, y) and the Fourier transform of *p*(*x*, *y*) are combined to encode the input. Mathematically, the field on input *T*(*x*, *y*; *z*_*c*_) due to *p*(*x*, *y*) is, besides some constant terms,

1 $$\begin{array}{l}[\Im {\{p(x,y)\}}_{{k}_{x}={k}_{0}x/f,{k}_{y}={k}_{0}y/f}\ast h(x,y;{z}_{c})]\exp (j{\omega }_{0}t)\\ \quad =[\tilde{p}({k}_{0}x/f,{k}_{0}y/f)]\ast h(x,y;{z}_{c})\exp (j{\omega }_{0}t)=P(x,y;2f+{z}_{c})\exp (j{\omega }_{0}t)\end{array},$$

where the definition of Fourier transform is

$$\Im {\{g(x,y)\}}_{{k}_{x},{k}_{y}}=\tilde{g}({k}_{x},{k}_{y})={\int }_{-\infty }^{\infty }{\int }_{-\infty }^{\infty }g(x,y)\exp (j{k}_{x}x+j{k}_{y}y)dxdy$$

with *k*_*x*_ and *k*_*y*_ denoting spatial frequencies. Symbol * denotes convolution between the two functions^21,26^ and $h(x,y;z)=\exp (-j{k}_{0}z)\frac{j{k}_{0}}{2\pi z}\exp \{\frac{-j{k}_{0}({x}^{2}+{y}^{2})}{2z}\}$ is the free space impulse response in Fourier optics^21,26^. Now, for the field on input *T*(*x*, *y*; *z*_*c*_) due to *q*(*x*, *y*), according to Fresnel diffraction, is

2 $$\begin{array}{l}[q(x,y)\ast h(x,y;{z}_{q}=2f+{z}_{c})]\exp [j({\omega }_{0}+{\rm{\Omega }})t]\\ \quad =Q(x,y;{z}_{q}=2f+{z}_{c})\exp [j({\omega }_{0}+{\rm{\Omega }})t]\end{array},$$

where we have assumed equal optical path length (OPL) for both $p(x,y)$ and $q(x,y)$ for simplicity, i.e., we let *z*_*q*_ = *z*_*q0*_ + *z*_*c*_ = 2 *f* + *z*_*c*_.

The total scanning field on the object, according to equations (1) and (2) is, therefore, given by

3 $$S(x,y;{z}_{c})=P(x,y;2f+{z}_{c})\exp (j{\omega }_{0}t)+Q(x,y;2f+{z}_{c})\exp [j({\omega }_{0}+{\rm{\Omega }})t],$$

and the field after the input transparency is *S*(*x*′, *y*′; *z*_*c*_)*T*(*x*′ + *x*, *y*′ + *y*; *z*_*c*_), where *x* = *x*(*t*) and *y* = *y*(*t*) represent the instantaneous 2-D position of the object during the action of *xy*-scanning. Finally, the photodetector (PD) gives the current output by spatially integrating the intensity:

4 $$\begin{array}{c}i(x,y;{z}_{c})\propto {\int }_{A}|S(x\text{'},y\text{'};{z}_{c})T(x\text{'}+x,y\text{'}+y;{z}_{c}){|}^{2}dx\text{'}dy\text{'}\\ ={\int }_{A}|\{P(x\text{'},y\text{'};2f+{z}_{c})\exp (j{\omega }_{0}t)+Q(x\text{'},y\text{'};2f+{z}_{c})\exp [j({\omega }_{0}+{\rm{\Omega }})t]\}\\ \times T(x\text{'}+x,y\text{'}+y;{z}_{c}){|}^{2}dx\text{'}dy\text{'},\end{array}$$

where *A* is the active area of the PD. After bandpass filtering of *i*(*x*, *y*; *z*_*c*_) at the heterodyne frequency Ω, the heterodyne current is

5 $${i}_{{\rm{\Omega }}}(x,y;{z}_{c})\propto {\rm{Re}}[{i}_{{\rm{\Omega }}p}(x,y;{z}_{c})\exp (j{\rm{\Omega }}t)],$$

where

6 $${i}_{\Omega p}(x,y;{z}_{c})={\int }_{A}{P}^{\ast }(x\text{'},y\text{'};2f+{z}_{c})Q(x\text{'},y\text{'};2f+{z}_{c})|T(x\text{'}+x,y\text{'}+y;{z}_{c}){|}^{2}dx\text{'}dy\text{'}]$$

is the current phasor, which contains the amplitude and phase information of *i*_Ω_(*x*, *y*; *z*_*c*_).

The phasor current above can be expressed in terms of correlation as follows:

7 $${i}_{{\rm{\Omega }}p}(x,y;{z}_{c})=P(x,y;2f+{z}_{c}){Q}^{\ast }(x,y;2f+{z}_{c})\otimes |T(x,y;{z}_{c}){|}^{2},$$

where correlation of *g*_1_(*x*, *y*) and *g*_2_(*x*, *y*) is defined as

$${g}_{1}(x,y)\otimes {g}_{2}(x,y)={\int }_{-{\rm{\infty }}}^{{\rm{\infty }}}{\int }_{-{\rm{\infty }}}^{{\rm{\infty }}}{g}_{1}^{\ast }(x\text{'},y\text{'}){g}_{2}(x+x\text{'},y+y\text{'})dx\text{'}dy\text{'}.$$

Taking the Fourier transform of equation (7), we have

$$\Im \{{i}_{{\rm{\Omega }}p}(x,y;{z}_{c})\}={\Im }^{\ast }\{P(x,y;2f+{z}_{c}){Q}^{\ast }(x,y;2f+{z}_{c})\}\Im \{|T(x,y;{z}_{c}){|}^{2}\}.$$

We can now define the optical transfer function (OTF) of the system as

8 $$\begin{array}{lcc}OTF({k}_{x},{k}_{y};{z}_{c}) & = & \Im \{{i}_{{\rm{\Omega }}p}(x,y;{z}_{c})\}/\Im \{|T(x,y;{z}_{c}){|}^{2}\}\\  & = & {\Im }^{\ast }\{P(x,y;2f+{z}_{c}){Q}^{\ast }(x,y;2f+{z}_{c})\}\end{array}.$$

So the output heterodyne current from the PD can be expressed as, using equation (8),

9 $$\begin{array}{l}{i}_{{\rm{\Omega }}}(x,y;{z}_{c})\propto {\rm{Re}}[{i}_{{\rm{\Omega }}p}\exp (j\Omega t)]\\ \quad =\mathrm{Re}[{\Im }^{-1}\{\Im \{|T(x,y;{z}_{c}){|}^{2}\}OTF({k}_{x},{k}_{y};{z}_{c})\}\exp (j{\rm{\Omega }}t)]]\end{array}.$$

The amplitude and phase of the above current can be extracted conveniently by a lock-in amplifier as shown in Fig. 1 and the two final outputs, the in-phase component *i*_*I*_(*x*, *y*; *z*_*c*_) and the quadrature component *i*_*Q*_(*x*, *y*; *z*_*c*_) are as follows:

10a $${i}_{I}(x,y;{z}_{c})={\rm{Re}}\,[{\Im }^{-1}\{\Im \{|T(x,y;{z}_{c}){|}^{2}\}OTF({k}_{x},{k}_{y};{z}_{c})\}]$$

10b $${i}_{Q}(x,y;{z}_{c})={\rm{I}}{\rm{m}}\,[{{\rm{\Im }}}^{-1}\{{\rm{\Im }}\{|T(x,y;{z}_{c}){|}^{2}\}OTF({k}_{x},{k}_{y};{z}_{c})\}].$$

A complex record of the coded or encrypted object can be constructed in a computer according to the following complex relation:

11 $$\begin{array}{l}{i}_{I}(x,y;{z}_{c})+j{i}_{Q}(x,y;{z}_{c})\\ \quad ={\Im }^{-1}\{\Im \{|T(x,y;{z}_{c}){|}^{2}\}OTF({k}_{x},{k}_{y};{z}_{c})\}={H}_{C}^{en}(x,y;{z}_{c})\end{array}.$$

${H}_{C}^{en}(x,y;{z}_{c})$ is called the encrypted hologram of the 3-D object, |*T*(*x*, *y*; *z*_*c*_)|^2^. It is clear from the above that the object intensity distribution, i.e., |*T*(*x*, *y*; *z*_*c*_)|^2^, is being processed and, therefore, the optical system is incoherent. The spectrum of the object is now processed or encrypted by the OTF given by equation (8).

The OTF in equation (8) can be expressed in terms of the two coding masks, *p*(*x*, *y*) and *q*(*x*, *y*), by using expressions *P*(*x*, *y*; 2*f* + *z*_*c*_) and *Q*(*x*, *y*; 2*f* + *z*_*c*_) from equations (1) and (2), respectively into equation (8). After some lengthy manipulations, we have

12 $$\begin{array}{l}OTF({k}_{x},{k}_{y};{z}_{c})={e}^{j\frac{{z}_{c}+2f}{2{k}_{0}}({k}_{x}^{2}+{k}_{y}^{2})}\\ \quad \quad \quad \quad \quad \quad \,\,\,\,\times \iint {p}^{\ast }(x\text{'},y\text{'})\tilde{q}(-\frac{{k}_{0}}{f}x\text{'}-{k}_{x},-\frac{{k}_{0}}{f}y\text{'}-{k}_{y}){e}^{j\frac{{k}_{0}}{f}(x{\text{'}}^{2}+y{\text{'}}^{2})}{e}^{j\frac{{z}_{c}+2f}{f}(x\text{'}{k}_{x}+y\text{'}{k}_{y})}dx\text{'}dy\text{'}\end{array}.$$

This *OTF* is able to record holographically the encrypted object, located at a distance of *z*_*c*_ + *f* distance away from Lens *L*, as indicated by the quadratic phase term, ${e}^{j\frac{{z}_{c}+2f}{2{k}_{0}}({k}_{x}^{2}+{k}_{y}^{2})}$, in front of the integral^26^. The remaining integral term is responsible for coding or encrypting the object, and the degree of encryption can be manipulated by masks *p*(*x*, *y*) and *q*(*x*, *y*). The overall effect is that we have an encrypted complex hologram, ${H}_{C}^{en}(x,y;{z}_{c})$, of object |*T*(*x*, *y*; *z*_*c*_)|^2^ according to equations (11) and (12).

### Decryption theory

The decryption process for recovering the object image |*T*(*x*, *y*; *z*_*c*_)|^2^ from the encrypted hologram ${H}_{C}^{en}(x,y;{z}_{c})$ is outline as follows. To begin with, we have assumed the *unity condition* given by *OTF* ^*^(*k*_*x*_, *k*_*y*_; *z*_*d*_) *OTF*(*k*_*x*_, *k*_*y*_; *z*_*d*_) = 1, where *z*_*d*_ is the decoding distance and ${z}_{c}\in {z}_{d}$, i.e., ${z}_{c}$ belongs to ${z}_{d}$. As such, it can be easily inferred from equation (11) that the original object |*T*(*x*, *y*; *z*_*c*_)|^2^ can be recovered by multiplying the Fourier transform of the encrypted data ${H}_{C}^{en}(x,y;{z}_{c})$ with the conjugate of the optical transfer function evaluated at  decoding distance *z*_*d*_ = *z*_*c*_, i.e.,

13 $$\begin{array}{l}{\Im }^{-1}\{\Im \{{H}_{c}^{en}(x,y;{z}_{c})\}OT{F}^{\ast }({k}_{x},{k}_{y};{z}_{d})|{z}_{d}={z}_{c}\}\\ \quad ={\Im }^{-1}\{\Im \{{|T(x,y;{z}_{c})|}^{2}\}OTF({k}_{x},{k}_{y};{z}_{c})OT{F}^{\ast }({k}_{x},{k}_{y};{z}_{c})\}\\ \quad ={\Im }^{-1}\{\Im \{{|T(x,y;{z}_{c})|}^{2}\}\}={|T(x,y;{z}_{c})|}^{2}\end{array}.$$

The function *OTF* ^*^(*k*_*x*_, *k*_*y*_; *z*_*c*_), which encapsulates the pair of masks *p*(*x*, *y*) and *q*(*x*, *y*), is needed in order to decrypt the information. To determine *OTF* ^*^(*k*_*x*_, *k*_*y*_; *z*_*c*_) (assuming, *p*(*x*, *y*), *q*(*x*, *y*), *z*_*c*_ are available) for recovering |*T*(*x*, *y*; *z*_*c*_)|^2^, we first obtain a pin hole hologram *H* ^*δ*^(*k*_*x*_, *k*_*y*_; *z*_*d*_ = *z*_*c*_) by scanning a pin hole with the system shown in Fig. 1 when the switch is at *K*_2_. From equation (11), it can be seen that the pin hole hologram can be derived by replacing the term |*T*(*x*, *y*; *z*_*c*_)|^2^ with the pin hole function denoted by *δ*(*x*, *y*; *z*_*d*_), i.e., |*T*(*x*, *y*; *z*_*c*_)|^2^ = *δ*(*x*, *y*; *z*_*d*_) with *z*_*c*_ = *z*_*d*_, resulting in a pin hole hologram given by

$$\begin{array}{l}{H}^{\delta }(x,y;{z}_{d})={i}_{I}(x,y;{z}_{d})+j{i}_{Q}(x,y;{z}_{d})={\Im }^{-1}\{\Im \{\delta (x,y;{z}_{d})\}OTF({k}_{x},{k}_{y};{z}_{d})\}\\ \quad ={\Im }^{-1}\{OTF({k}_{x},{k}_{y};{z}_{d})\}\end{array},$$

thus giving

$$OT{F}^{\ast }({k}_{x},{k}_{y};{z}_{d})=\Im {\{{H}^{\delta }(x,y;{z}_{d})\}}^{\ast }.$$

Hence, *OTF* ^*^(*k*_*x*_, *k*_*y*_; *z*_*d*_), to be used in equation (13), is derived from the pin hole hologram. From the above equation and equation (13), we can infer that the encrypted image can be recovered by convolving the encrypted hologram, ${H}_{C}^{en}(x,y;{z}_{c})$, with the pinhole hologram, *H* ^*δ*^(*x*, *y*; *z*_*c*_), i.e., it can be shown readily that

14 $$\begin{array}{l}{H}^{\delta }(x,y;{z}_{d}={z}_{c})\otimes {H}_{C}^{en}(x,y;{z}_{c})\\ \quad ={\Im }^{-1}\{\Im \{{|T(x,y;{z}_{c})|}^{2}\}OTF({k}_{x},{k}_{y};{z}_{c})OT{F}^{\ast }({k}_{x},{k}_{y};{z}_{c})\}\\ \quad =|T(x,y;{z}_{c}){|}^{2}\end{array},$$

where $\otimes$ denotes correlation involving *x* and *y* ^21,26^. Equation (14) expresses the essential feature of the proposed technique succinctly. We simply obtain two holograms, ${H}_{C}^{en}(x,y;{z}_{c})$ and ${H}^{\delta }(x,y;{z}_{c})$, experimentally for the overall encryption and decryption process.

In this Section, we have discussed the encryption and decryption of a planar image. In the next Section we shall describe how our proposed method can be extended to optical cryptography of 3-D object images with the incorporation of biometrics information.

## Optical cryptography with biometrics on 3-D object images

To start with, we would like to explain the extension of our proposed method to biometric optical cryptography.

### Optical cryptography with biometrics

In order to allow for biometric authentication, in the encryption system the *encryption key q*(*x*, *y*), is derived from the product of the message sender’s fingerprint *FP*_1_(*x*, *y*), and a random phase mask *RPM*_1_(*x*, *y*), i.e., *q*(*x*, *y*) = *FP*_1_(*x*, *y*) *RPM*_1_(*x*, *y*). This can be realized optically if we stack two spatial light modulators together, one for the fingerprint and the other for the phase mask. The other mask, *p*(*x*, *y*), again can be treated the same way as *p*(*x*, *y*) = *FP*_2_(*x*, *y*) *RPM*_2_(*x*, *y*), where *FP*_2_(*x*, *y*) is the message receiver’s fingerprint, and *RPM*_2_(*x*, *y*) is another random phase mask. *RPM*_1_(*x*, *y*) and *RPM*_2_(*x*, *y*) are two independent random functions that allow the system to be of high security. Both random phase masks can be preset in the encryption and decryption systems, so that the message sender and the message recipient do not have to remember or keep them to avoid the lost-key situation.

To further enhance the security of the cryptographic system, the message sender’s fingerprint information *q*(*x*, *y*) (hereafter referred to as the 1^st^ key) is shared in advance with the message receiver. The 2^nd^ key *p*(*x*, *y*) is only sent to the sender when the recipient request the sending of an encrypted image. With this additional measure, the encrypted hologram cannot be decrypted even if one possessed the 1^st^ key from the sender through theft or other illegitimate means.

Since fingerprints, *FP*_1_(*x*, *y*) and *FP*_2_(*x*, *y*), are of amplitude information, the use of *p*(*x*, *y*) = *FP*_2_(*x*, *y*) *RPM*_2_(*x*, *y*) and *q*(*x*, *y*) = *FP*_1_(*x*, *y*) *RPM*_1_(*x*, *y*) in Eq. (12) will not meet the unity condition as the obtained *OTF* will have amplitude distribution. To overcome the issue, let us work on Eq. (14) by noticing that the OTF given by Eq. (12) is complex in general and we could write *OTF*(*k*_*x*_, *k*_*y*_; *z*_*c*_) = A(*k*_*x*_, *k*_*y*_; *z*_*c*_)*e* ^*jθ*^((*k*_*x*_, *k*_*y*_; *z*_*c*_)). In light of this, by taking the Fourier transform of Eq. (14), we have

$$\Im \{{H}^{\delta }(x,y;{z}_{d}={z}_{c})\otimes {H}_{C}^{en}(x,y;{z}_{c})\}=\Im \{|T(x,y;{z}_{c}){|}^{2}\}{{\rm{A}}}^{2}({k}_{x},{k}_{y};{z}_{c}),$$

which can be manipulated to give

15 $$|T(x,y;{z}_{c}){|}^{2}={\Im }^{-1}\{\Im \{{H}^{\delta }(x,y;{z}_{d}={z}_{c})\otimes {H}_{C}^{en}(x,y;{z}_{c})\}/{{\rm{A}}}^{2}({k}_{x},{k}_{y};{z}_{c})\}.$$

Under this situation, we need to know the encrypted hologram, ${H}_{C}^{en}(x,y;{z}_{c})$, the pinhole hologram, *H* ^*δ*^(*x*, *y*; *z*_*c*_) as well as ${{\rm{A}}}^{2}({k}_{x},{k}_{y};{z}_{c})$ in order to perfectly decrypt $|T(x,y;{z}_{c}){|}^{2}$. The knowledge of A(*k*_*x*_, *k*_*y*_; *z*_*c*_) can be obtained experimentally through the pin hole hologram *H* ^*δ*^(*x*, *y*; *z*_*d*_ = *z*_*c*_) as

16 $${\rm{A}}({k}_{x},{k}_{y};{z}_{c})=|\Im \{{H}^{\delta }(x,y;{z}_{d}={z}_{c})\}|=|OTF({k}_{x},{k}_{y};{z}_{c})|.$$

Hence with the inclusion of the term ${{\rm{A}}}^{2}({k}_{x},{k}_{y};{z}_{c})$ in order to perfectly decrypt the image, we call this process as the compensation process.

### Optical cryptography on 3-D object images

We model a 3-D object images as a collection of planar objects as $\sum _{m=1}^{M}|{T}_{m}(x,y;{z}_{m}){|}^{2}$, where |*T*_*m*_(*x*, *y*; *z*_*m*_)|^2^ is the 2-D intensity distribution at various axial depths, *z*_*m*_, i.e., it is the sectional images of the 3-D object. Hence, for 3-D objects, ${H}_{C}^{en}(x,y)$ becomes, according to Eq. (11),

17 $${H}_{C}^{en}(x,y)={\Im }^{-1}\{\sum _{m=1}^{M}\{\Im [{|{T}_{m}(x,y;{z}_{m})|}^{2}]OTF({k}_{x},{k}_{y};{z}_{m})\}\}.$$

So in the case of encrypting a 3-D object, we have many decoding distances *z*_*m*_. To extract or decrypt a specific transverse plane at *z*_*k*_, where *k* = [1, *M*], we first record the pinhole hologram *H* ^*δ*^(*x*, *y*; *z*_*k*_) at *z*_*d*_ = *z*_*k*_ in the decryption stage, and then correlate it with the encrypted hologram as

18 $$\begin{array}{l}{H}^{\delta }(x,y;{z}_{d}={z}_{k})\otimes {H}_{C}^{en}(x,y)\\ ={\Im }^{-1}\{\sum _{m=1}^{M}\{\Im [{|{T}_{m}(x,y;{z}_{m})|}^{2}]OTF({k}_{x},{k}_{y};{z}_{m})\}OT{F}^{\ast }({k}_{x},{k}_{y};{z}_{k})\}\\ ={|{T}_{k}(x,y;{z}_{k})|}^{2}+{\Im }^{-1}\{\sum _{m\ne k}^{M}\{\Im [{|{T}_{m}(x,y;{z}_{m})|}^{2}]OTF({k}_{x},{k}_{y};{z}_{m})\}OT{F}^{\ast }({k}_{x},{k}_{y};{z}_{k})\}\end{array}.$$

From equation (18), the first term of the expression on the right-hand-side is the full recovery of the sectional image of the original object image at *z*_*k*_, while the rest of the terms are defocused noise. By repeating the above process from *k* = 1 to *k* = *M*, all the sectional images of the object can be recovered. We will show experimental results in the next section.

## Experimental results

**Figure 2:**
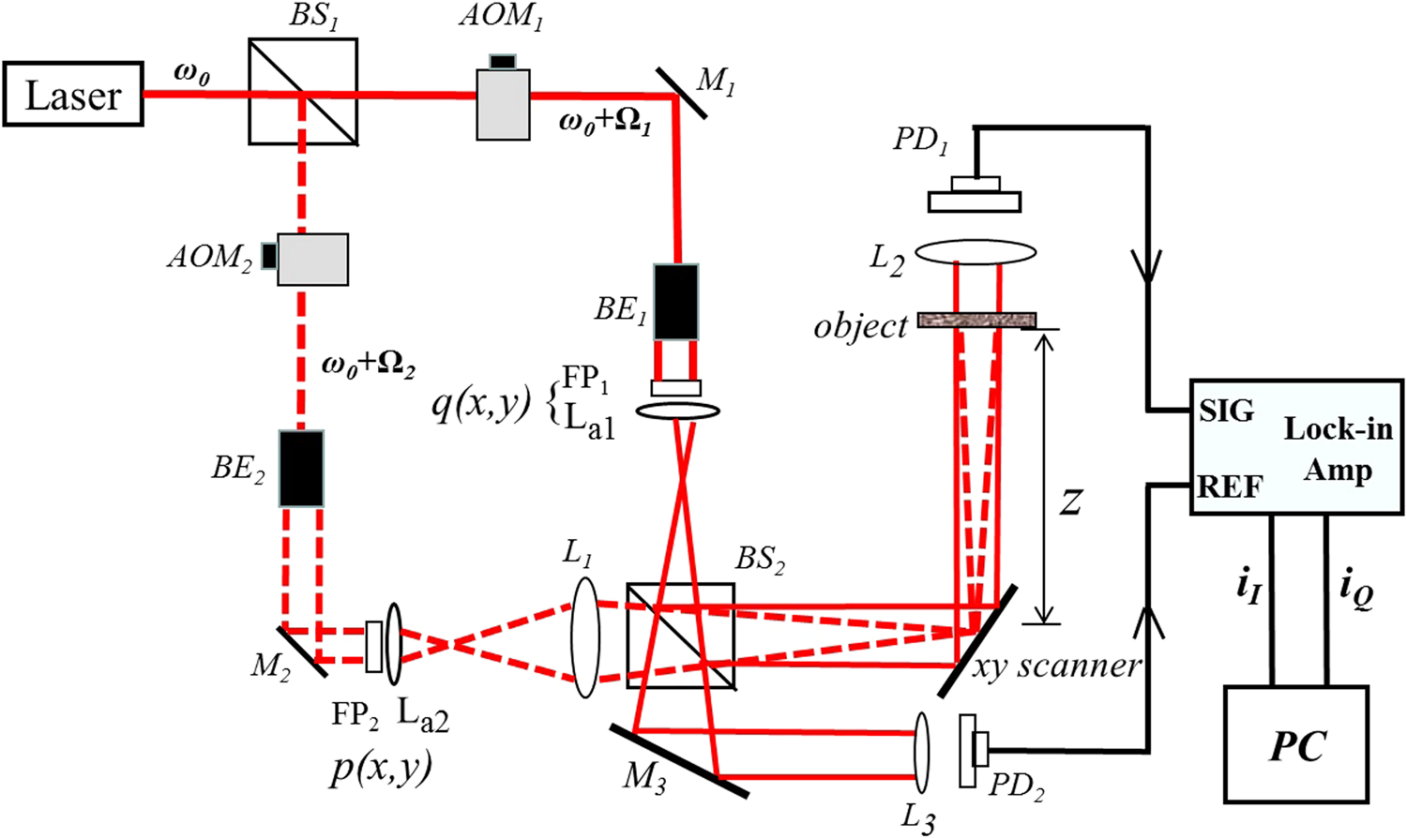
Experimental setup for the optical cryptosystem (*BS*_1_,_2_: beam splitter, *L*_1_: Fourier transform lens, *L*_2_: a lens for collecting all the optical energy onto photodetector *PD*_1_, which gives the scanned heterodyne signal. The output of photodetector *PD*_2_ gives a heterodyne frequency as a reference signal to the lock-in amplifier).

In order to verify the feasibility of the proposed optical cryptosystem, the proof-of-principle experiment has been implemented. The experimental setup is shown in Fig. 2.

A laser at frequency ω_0_ is used to split into two beams. The laser’s wavelength is 632.8 nm with laser power of 15 mW. The two masks *q*(*x*, *y*) and *p*(*x*, *y*) are illuminated by the laser at frequency ω_0_ + Ω_1_ and ω_0_ + Ω_2_, respectively. Two acousto-optic modulators, AOM_1_ and AOM_2_, operating at frequencies Ω_1_ and Ω_2_, are used to upshift the laser beam frequency at ω_0_, to ω_0_ + Ω_1_ and ω_0_ + Ω_2_, respectively. The heterodyne frequency is at (Ω_1_ − Ω_2_)/2π = 25 kHz_._ The two masks *p*(*x*, *y*) and *q*(*x*, *y*) in general can be implemented by SLMs displaying the fingerprint images and random phase masks. However, owing to the current resource limitation in our laboratory, we simply make a proof-of-concept study with two lenses (*L*_*a*1_ and *L*_*a*2_ with focal length of *f*_a1_ = 75.6mm and *f*_a2_ = 150 mm, respectively) generating quadratic phase modulation instead of random phases. Hence, *q*(*x*, *y*) = *FP*_1_(*x*, *y*)exp[jπ(*x* ^2^ + *y* ^2^)/(λ*f*_a1_)] and *p*(*x*, *y*) = *FP*_2_(*x*, *y*)exp[jπ(*x* ^2^ + *y* ^2^)/(λ*f*_a2_)]. Again *FP*_1_(*x*, *y*) is the message sender’s fingerprint, and *FP*_2_(*x*, *y*) is the message receiver’s fingerprint. In the experiments, the fignerprints are in the form of transparencies. BE_1_ and BE_2_ are two expanders so that the output of them will give uniform plane waves illuminating the two masks *q*(*x*, *y*) and *p*(*x*, *y*). The size of fingerprint images is a transparency of about 1.4 cm × 1.8 cm, and the focal length of Fourier transform lens *L*_1_ is 300 mm.

**Figure 3:**
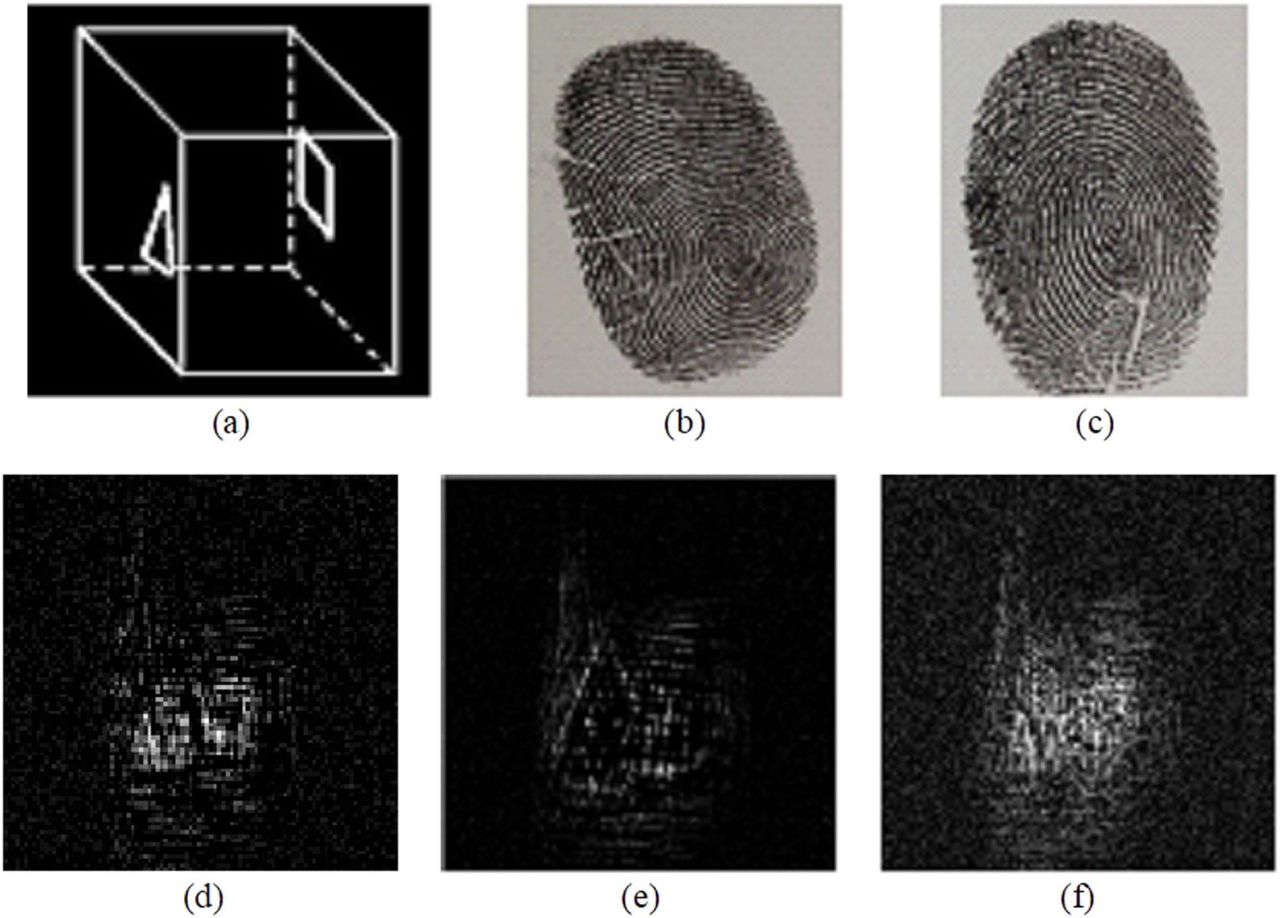
(**a**) 3-D object to be encrypted, (**b**) message sender’s fingerprint (**c**) message receiver’s fingerprint, (**d**) Real part of encrypted complex hologram ${H}_{C}^{en}(x,y)$, (**e**) Imaginary part of encrypted complex hologram ${H}_{C}^{en}(x,y)$, and (**f**) intensity distribution of the encrypted complex hologram.

Figure 3(a) shows a 3-D object to be encrypted, consisting of a triangle and a square separated by 5.5 cm along the depth of the object. “Δ” is located at *z* = 30 cm and “□” at *z* = 35.5 cm. The 3-D object is approximately 1 × 1 × 5.5 cm^3^ and is transmissive on an opaque background with an opening linewidth of about 100 μm. Figure 3(b) shows the message sender’s fingerprint. Figure 3(c) shows the message receiver’s fingerprint, and Fig. 3(d),(e),(f) show the real part, the imaginary part and the intensity of the encrypted complex hologram, respectively, which are generated from the in-phase and quadrature-phase signals [see Eq. (11)]. It can be seen from Fig. 3(f) that the pattern of the object is seriously disturbed and no useful information about the original object can be identified.

**Figure 4:**
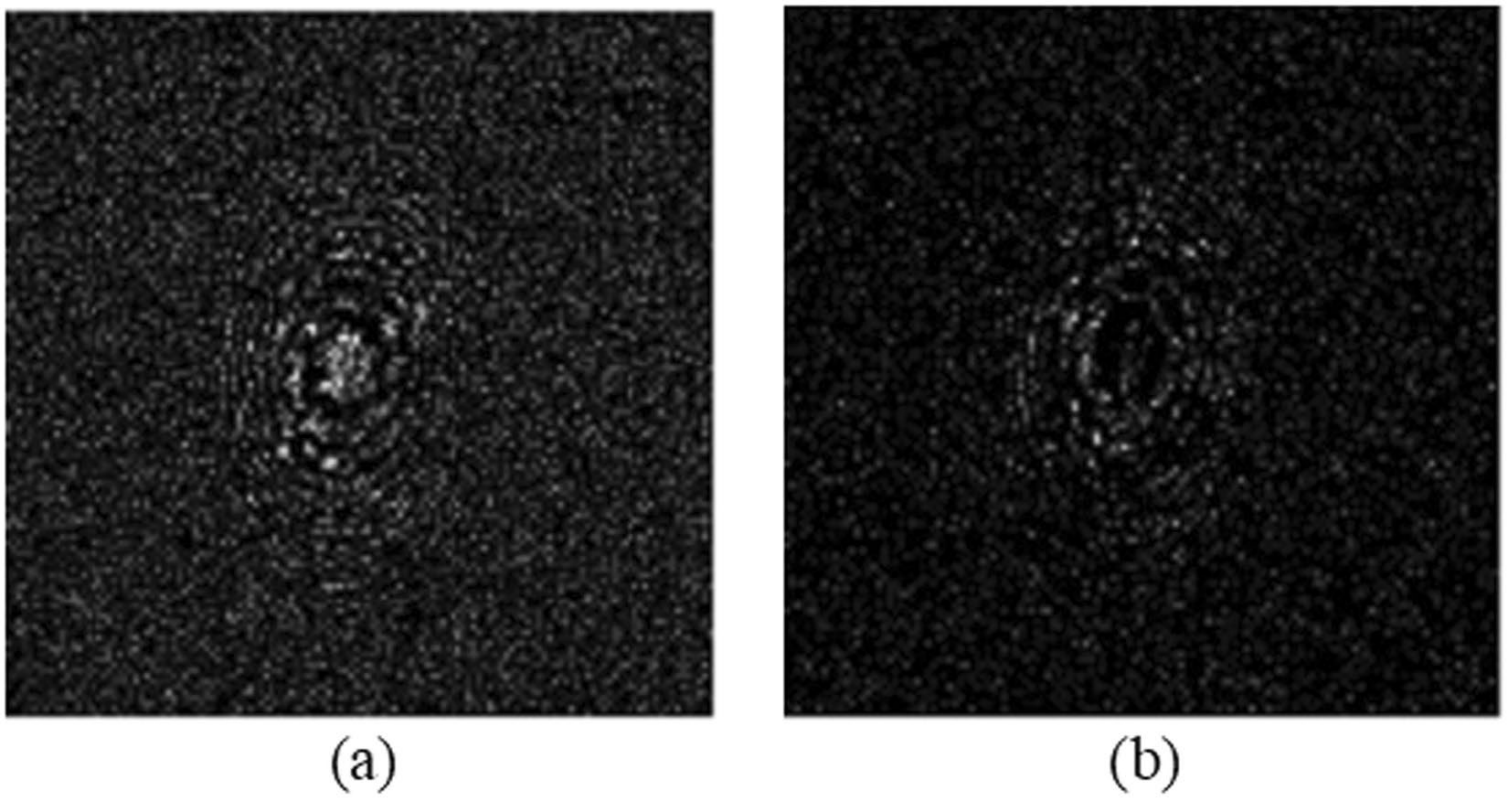
(**a**) Real part, and (**b**) imaginary part of the pinhole hologram measured at decoding distance z_*d*1_ = 30 cm, i.e., *H* ^*δ*^(*x*, *y*; *z*_*d*1_ = 30 cm).

**Figure 5:**
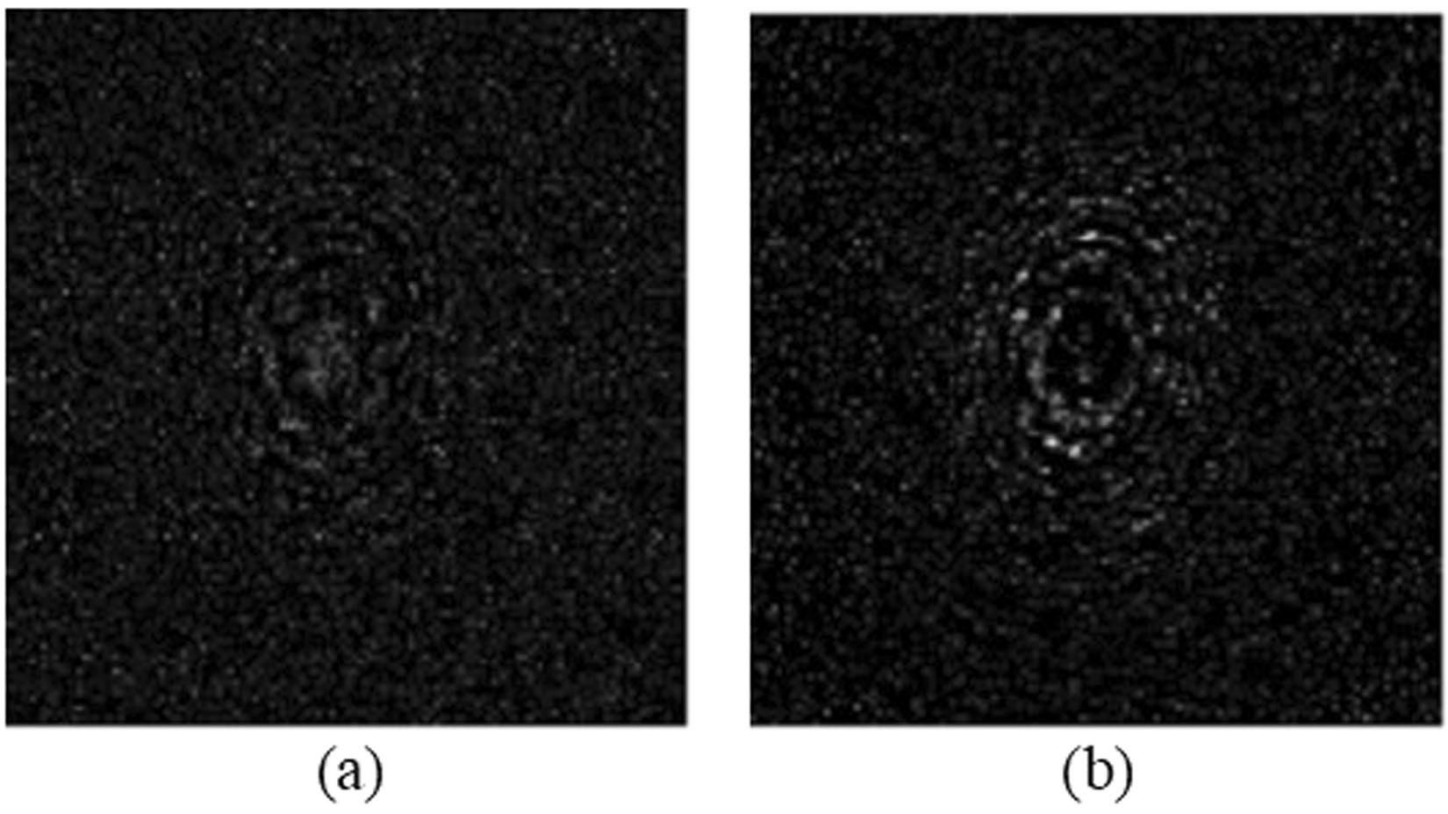
(**a**) Real part, and (**b**) imaginary part of the pinhole hologram measured at decoding distance *z*_*d*2_ = 35.5 cm, i.e., *H* ^*δ*^(*x*, *y*; *z*_*d*2_ = 35.5 cm).

From the developed decryption theory, in the decryption stage, we need to find the pin hole hologram. In light of it, we use a pinhole of 0.28 mm in diameter to replace the object and record the pinhole holograms at the corresponding decoding distances of z_*d*1_ = 30 cm and z_*d*2_ = 35.5 cm. In this case, the message sender’s fingerprint (see Fig. 3b) has been sent to the decryption stage and the message receiver’s fingerprint [see Fig. 3(c)] is the decryption key. Each pinhole hologram at different decoding distances becomes the decrypting hologram for that distance as shown in Eq. (18). The real and imaginary parts of the pin holograms for z_*d1*_ = 30 cm and z_*d2*_ = 35.5 cm are shown in Figs 4 and 5, respectively.

**Figure 6:**
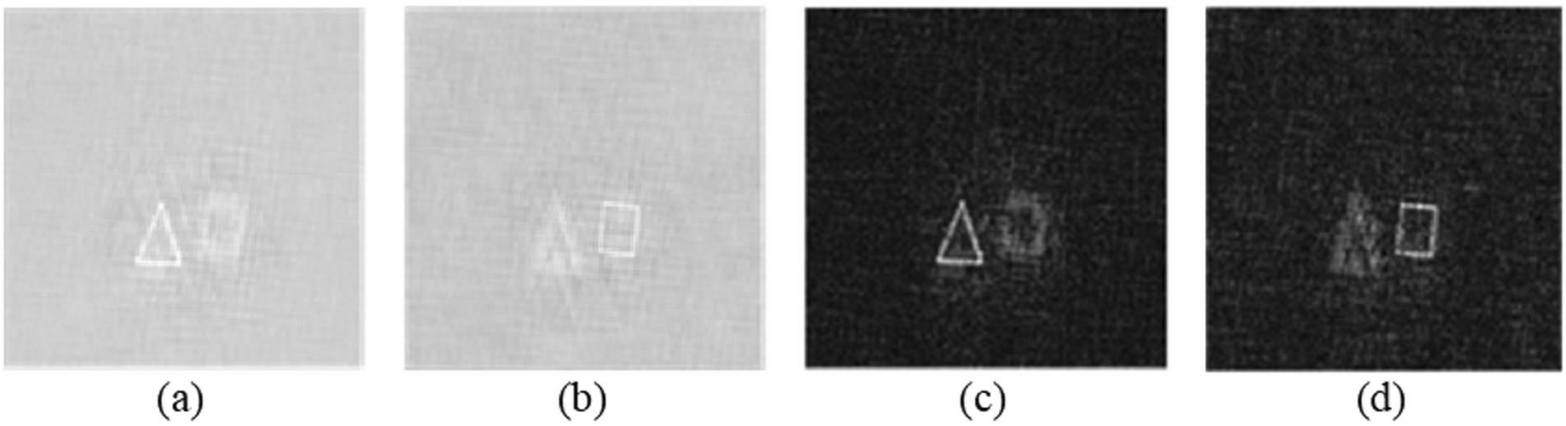
Decrypted sectional images using the pinhole holograms measured at (**a**) z_*d*1_ = 30 cm and (**b**) z_*d*2_ = 35.5 cm for unprocessed decrypted images when the unity condition is not met for fingerprint images, and at (**c**) z_*d*1_ = 30 cm and (**d**) z_*d*2_ = 35.5 cm for processed decrypted images.

Figure 6 shows the decrypted sectional images at the decoding distances z_*d*1_ = 30 cm and z_*d*2_ = 35.5 cm. For Fig. 6(a),(b), since the fingerprint images do not satisfy the unity condition, i.e., *OTF* ^*^(*k*_*x*_, *k*_*y*_; *z*_*d*_ = *z*_*c*_)*OTF*(*k*_*x*_, *k*_*y*_; *z*_*c*_) ≠ 1, the effect of low contrast of the decrypted images is obvious. In obtaining Fig. 6(a),(b), we simply correlate the pin hole hologram, ${H}^{\delta }(x,y;{z}_{d}={z}_{c})$, with the encrypted hologram, ${H}_{C}^{en}(x,y;{z}_{c})$. Figures 6 (c),(d) show the processed decrypted image according to Eq. (15) to overcome the fact that the unity condition is not met for fingerprint images, in that ${{\rm{A}}}^{2}({k}_{x},{k}_{y};{z}_{c})$, derived from the pin hologram hologram [see Eq. (16)], is used to perform the compensation process we discussed in Eq. (15). Note that pattern “Δ” is focused at the location of *z*_*d*1_ = 30 cm and the decrypted image is blurred elsewhere, corresponding to out-of-focus haze in 3-D imaging. Similarly, pattern “□” is focused at the location of *z*_*d*2_ = 35.5 cm with out-of-focus haze around the focused image. Hence, we have demonstrated that 3-D object can be encrypted and its sectional images can be decrypted in the proposed cryptosystem.

**Figure 7:**
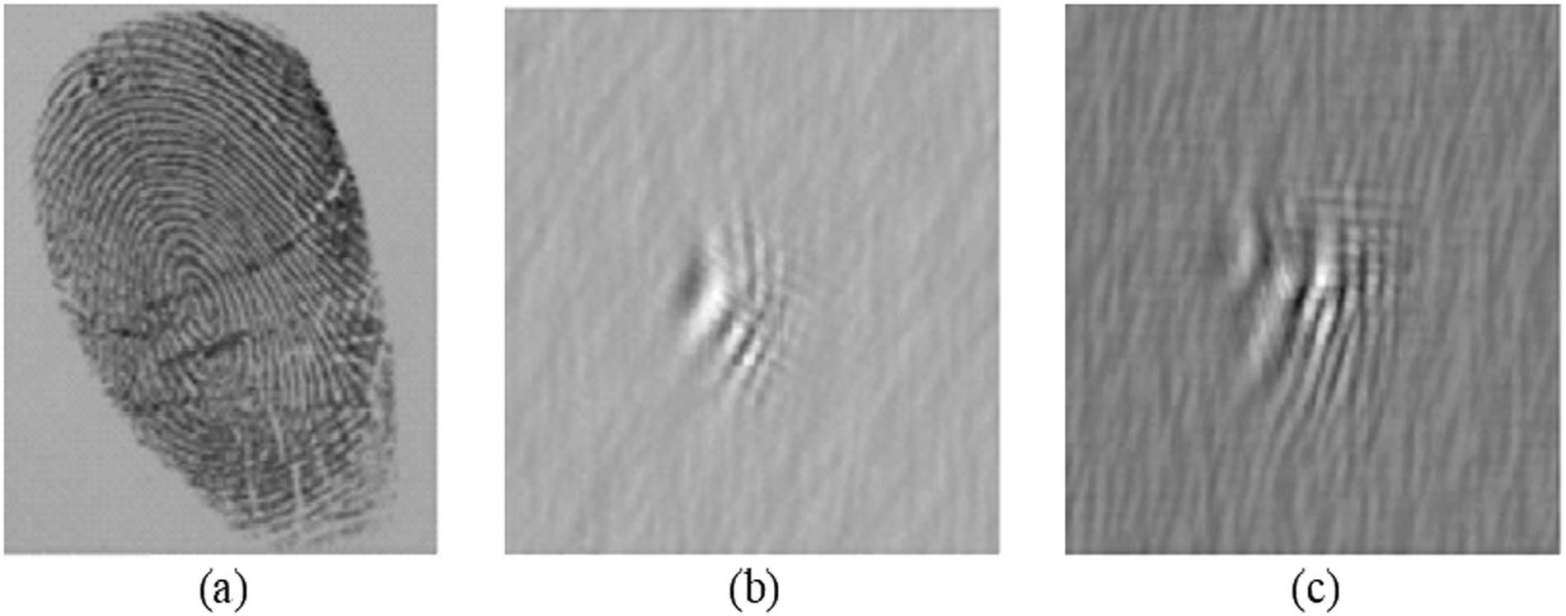
(**a**) Fingerprint as wrong decryption key, the decrypted images using the pinhole holograms generated by the wrong decryption key at the position of (**b**) *z*_*d*1_ = 30 cm, and (**c**) *z*_*d*2_ = 35.5 cm.

We have also examined experimentally the proposed system by using different fingerprints for decryption. In the experiment, the encryption system and encryption parameters are exactly the same as before. But, in the decryption process, different fingerprint image is used along with Lens *L*_*a*2_ taken away. Figure 7(a) shows the wrong fingerprint image used in the decryption system. Figure 7(b),(c) show the decrypted images using the pinhole holograms generated by the wrong fingerprint at two positions of *z*_*d*1_ = 30 cm and *z*_*d*2_ = 35.5 cm, respectively. We notice that although the decoding distance is correct, the object cannot be correctly decrypted and reconstructed because the wrong fingerprint key generates the wrong pinhole holograms for decryption. The original secret image cannot be deciphered even the encryption key is known by attackers. As a result, high security against illegal attacks can be obtained by the proposed cryptosystem.

We would also like to make a brief evaluation and assessment on the vulnerability of our method towards plaintext attacks. From Eq. (11) we can infer that theoretically, the encryption key will be deduced based on a pair of known, planar images $|{T}_{1}(x,y;{z}_{c}){|}^{2}$ and $|{T}_{2}(x,y;{z}_{c}){|}^{2}$, and their encrypted holograms ${H}_{C1}^{en}(x,y,{z}_{c})$ and ${H}_{C2}^{en}(x,y,{z}_{c})$. Both holograms are assumed to be encrypted at the same distance ${z}_{c}$. The process of deducing the encryption key is outlined as follows. First we compute the difference between the two encrypted holograms, each represented with Eq. (11), as

19 $$\begin{array}{lll}{H}_{diff} & = & {H}_{C1}^{en}(x,y,{z}_{c})-{H}_{C2}^{en}(x,y,{z}_{c})\\  & = & {\Im }^{-1}\{\Im \{|{T}_{1}(x,y;{z}_{c}){|}^{2}\}OTF({k}_{x},{k}_{y};{z}_{c})\}-{\Im }^{-1}\{\Im \{|{T}_{2}(x,y;{z}_{c}){|}^{2}\}OTF({k}_{x},{k}_{y};{z}_{c})\}\end{array}.$$

Next we apply Fourier transform to both sides of the above equation, which results in

20 $$\Im \{{H}_{diff}\}=\{\Im \{|{T}_{1}(x,y;{z}_{c}){|}^{2}\}OTF({k}_{x},{k}_{y};{z}_{c})\}-\Im \{|{T}_{2}(x,y;{z}_{c}){|}^{2}OTF({k}_{x},{k}_{y};{z}_{c})\}.$$

Rearranging the terms in Eq. (20), the encryption function can be deduced, as given by

21 $$OTF({k}_{x},{k}_{y};{z}_{c})=\frac{\Im \{{H}_{diff}\}}{\Im \{{|{T}_{1}(x,y;{z}_{c})|}^{2}\}-\Im \{{|{T}_{2}(x,y;{z}_{c})|}^{2}\}}.$$

As such if the intensity images are accidentally exposed through theft or other hacking activities, there is a good chance that the encryption key will also be deduced with Eqs (19–21). However, this kind of attack is difficult, if not impossible to achieve in practice, as the object that is being encrypted is directly converted into the encrypted hologram with the proposed system. In another words, the intensity image of the object is never recorded physically, and hence unknown even to the person who is performing the encryption.

**Figure 8:**
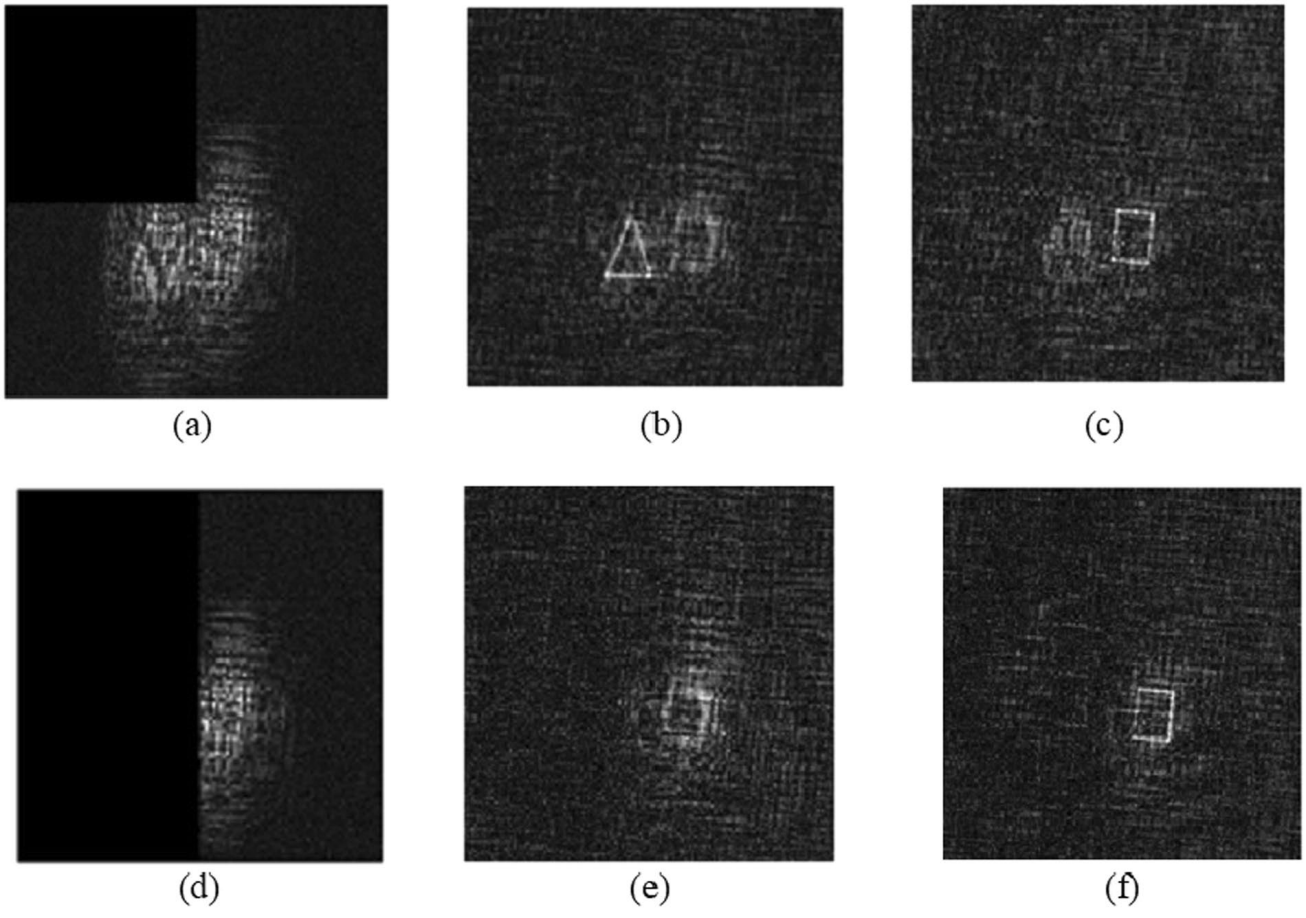
Occlusion results for the decrypted images with different degrees of occlusion. (**a**) with 25% occlusion (blanking the second quadrant of the hologram), (**b**) corresponding decrypted image from (**a**) at decoding distance *z*_1_, (**c**) corresponding decrypted image from (**a**) at decoding distance *z*_2_, (**d**) with 50% occlusion, i.e., blanking half (left side) of the hologram, (**e**) corresponding decrypted image from (**d**) at decoding distance *z*_1_, and (**f**) corresponding decrypted image from (**d**) at decoding distance *z*_2_.

While resistance to occlusion is, in general, not mandatory in encryption, in any case, we have performed a couple of cases rather than exhaustive investigation to provide some indication of the robustness of the proposed technique. According to our actual situation in the experiment, the decrypted images at decoding distance z_1_ = 30 cm and z_2_ = 35.5 cm are shown in Fig. 6(c),(d)). We define the mean square error (MSE) as

22 $$\mathrm{MSE}(z)=\frac{1}{M\times N}\sum _{i=1}^{M}\sum _{j=1}^{N}{[|{I}_{o}(i,j;z)-{I}_{r}(i,j;z)|]}^{2},$$

where *I*_*o*_(*i*, *j*; *z*) is a part of the decrypted image from the encrypted hologram without occlusion, *I*_*r*_(*i*, *j*; *z*) is a part of the decrypted image from the occluded encrypted hologram with *z* = *z*_1_ or *z*_2_ based on the decoding distance; (*i*, *j*) denotes pixel positions. (*M* × *N*) denotes the total number of pixels of the image we have selected on the reconstruction plane. In our calculation, we have used *M* = *N* = 64. Figure 8 shows the results for two kinds of occlusion.

When one-fourth of the encrypted hologram occluded at the top left corner (Fig. 8(a)), the calculated MSE(*z*_1_) and MSE(*z*_2_) values between the decrypted images without occlusion (Fig. 6(c),(d)) and the corresponding decrypted images with occlusion (Fig. 8(b),(c)) using all the correct keys are 0.264 and 0.199, respectively. It is shown that the decrypted images using the pinhole holograms at z_1_ and z_2_ can be recognized in the case of 25% occlusion of the encrypted hologram. When half of the encrypted hologram is occluded (Fig. 8(d)), the corresponding decrypted images with occlusion have the MSE values of MSE(*z*_1_) = 3.478 and MSE(*z*_2_) = 0.195, respectively for Fig. 8e,f). In this case, we observe that the object (triangle) cannot be decrypted at the decoding distance z_1_ in the case of 50% occlusion because most of the hologram of the “triangle” has been occluded. But at the decoding distance z_2_, the decrypted image of the “square” can be recognized but with some errors.

## Concluding remarks

We have proposed a cryptosystem for 3-D object images based on the optical heterodyne technique and biometric fingerprint keys. With our proposed method, a 3-D multi-depth object image can be encrypted into a complex encrypted hologram. Subsequently, the 3-D object image can be recovered from the encrypted hologram by correlating the encrypted hologram with a set of pinhole holograms, each located at a specific depth plane. We have applied the optical cryptosystem we have built to encrypt and decrypt 3-D object images. When the correct biometric keys are available, all the sectional images are recovered from the encrypted hologram with only mild defocused noise, and practically free from speckle noise. If the incorrect biometric keys are presented, the decrypted images are completely different from the original ones. As a concluding remark, our proposed method has successfully extended conventional optical scanning cryptography (OSC)^25^ to biometric cryptography of 3-D object images. We have also enhance security against illegal attacks with a double key arrangement, whereby the key representing the fingerprint of the recipient is passed to the sender only when an encrypted image is requested by the recipient.
